# Impacts of heat stress under oceanic climate on fertility and reproductive physiology of dairy cows subjected to hormonal synchronization

**DOI:** 10.1007/s00484-025-02969-6

**Published:** 2025-07-22

**Authors:** E. N. Martínez, C. Castillo, L. Avendaño-Reyes, J. Hernández, J. L. Benedito, A. Rico, P. Garcia, R. Muiño

**Affiliations:** 1 Departament of Animal Pathology, Campus Terra-IBADER, University of Santiago de Compostela, Lugo, Spain; 2 Institute of Agricultural Sciences, Autonomous University of Baja California, Mexicali, Mexico; 3 Meira Veterinary Center, Galicia, Spain

**Keywords:** Temperature-humidity index, Dairy cattle, Oceanic climate, Heat stress, Modified double-ovsynch

## Abstract

Regions with oceanic climates are experiencing unprecedented climatic changes. This study assesses the presence of heat stress in these regions and its impact on reproductive in Holstein cows using the Temperature-Humidity Index (THI), a measure of thermal stress. Data was collected from 159 Holstein cows across experiments. Cows underwent a modified double-ovsynch synchronization protocol, while 96 cows were inseminated based on observed estrous. Cows were examined for body temperature, follicle and uterus examination, and serum progesterone concentrations to evaluate the impact of heat stress. The results of cows pregnancy rate in double-ovsynch synchronized cows were 48.5% and 43.3% in summer and winter seasons, respectively. While the pregnancy rate in non-synchronized cows were 29.5% and 32.7% in summer and winter season, respectively. Elevated THI levels (THI ≥ 72) have adversely effects on reproductive physiology, reduced estrous expression, decreased uterine blood flow, and altered progesterone concentrations. Mild heat stress during summer months in oceanic climate negatively impacted reproductive efficiency in dairy cows, for adaptive management strategies. The double-ovsynch protocol effectively stabilized fertility across seasons, demonstrating its value in improving reproductive outcomes under varying thermal conditions.

## Introduction

Certain countries have regions with an oceanic climate (McKnight and Hess 2000; Millison 2019), characterized by frequent cloud cover, regular rainfall, low-lying clouds, and weather fronts. Thunderstorms are rare due to limited interaction between hot and cold air masses. Precipitation mainly falls as rain, with occasional snowfall in winter, particularly in high-latitude “subpolar oceanic” regions. Severe weather events are uncommon (Scholarly Community Encyclopedia 2022). This study was conducted in a region with an oceanic climate. To date, no studies have assessed the THI’s impact on dairy cattle reproductive efficiency in such climates. In this region, 2023 marked the hottest year since 1961, with a peak temperature of 33.1ºC (Meteogalicia 2023). This presents an opportunity to study Holstein cows’ adaptive capacity in oceanic climates and identify measures to mitigate welfare and milk yield impacts. Dairy cattle, especially lactating Holstein cows, are highly affected by heat due to their high metabolism, which generates internal heat (Cartwright et al. 2023).

Lactating cattle have a thermal comfort range of −0.5° C to 20° C and 40%−80% relative humidity (Jhonson 1989; Hahn 1999; Lees et al. 2019). Above 21° C, evaporative mechanisms are activated, but high humidity reduces cooling efficiency (Bohmanova 2006). The Temperature-Humidity Index (THI), Mader et al. (2006) assesses thermal stress. Values below 72 indicate no stress, 72–78 mild stress, 79–88 moderate stress, 89–99 severe stress, and above 99 death (Armstrong 1994). Heat stress affects reproduction, reducing estrus duration and expression, impairing oocyte quality (Edwards et al. 2005; Schrock et al. 2007; Andreu-Vázquez et al. 2010; Shaarawy et al. 2024), and early embryo development (Ealy et al. 1993). It also reduces uterine blood flow (Gwazdauskas et al.^1973^; Roman-Ponce et al. 1978), disrupts hormones (Ronchi et al. 2001; Roth et al. 2001a, b; Shaarawy et al. 2024), and slows fetal growth (Tao and Dahl 2013).

Double-ovsynch (DO) protocols improve fertility in heat-stressed cows. Dirandeh et al. (2015) found that DO increased ovulation and pregnancy rates in heat-stressed lactating cows. Similarly, Li et al. (2023) reported higher fertility in primiparous Holsteins with THI > 73. These findings highlight the importance of tailored synchronization protocols in challenging thermal environments.

The main objectives of this study are to investigate whether temperature and humidity index (THI) reach stress-inducing levels for dairy cattle under oceanic climate conditions, as well as to analyze fertility rates and reproductive parameters in cattle subjected and not to hormonal synchronization under heat stress conditions, as well as between winter and summer seasons.

## Materials and methods

All standards for animal handling and care were strictly followed, as indicated in the Spanish Regulations (RD 53/2013), legal provision number 1337, and the European regulation of animals for scientific purposes (Council of Europe, Directive 2010/63/EU). This study was also authorized by the Bioethics Committee of the University of Santiago de Compostela, Spain, according to the relevant Spanish Regulations.

### Animals and housing

This trial was conducted on a commercial dairy farm located in north-west Spain from January to March 2023 and from July to September 2023. The lactating herd consisted of 227 Holstein dairy cows with an average milk production of 10,675 kg (4.0% fat, 3.3% protein). The herd was housed in barns with an E-W orientation and open ventilation; a mechanical fan system was installed in the ceiling and was automatically activated when the ambient temperature reached 23 °C. If needed, the farm manager could manually activate the system. Animals were housed in free-stall barns bedded with sand and all of them were under the same feeding and handling conditions.

Cows were fed a total mixed ration (TMR) consisting of corn silage, grass silage, and barley straw as forage, with a concentrate formulated from corn and soybean meal according to the NRC (2001) to meet the lactation requirements. Lactating cows were milked three times per day by a milking robot at different hours.

### Reproductive management

Each week, cows at 64 ± 5 days in milk (DIM) were stratified by parity (primiparous *vs* multiparous, limited to second calving), body condition score (2.5–3.5) (Edmonson et al. 1989), and absence of postpartum pathologies. These animals were then randomly assigned to two groups for first insemination: 1) G-A (n = 63), a group subjected to an ovulation synchronization and timed artificial insemination (TAI) protocol (double-ovsynch), and 2) G-B (n = 96), a group consisting of non-synchronized animal (64 ± 5 DIM, BCS between 2.5–3.5 (Edmonson et al. 1989)), free of postpartum pathologies and including both primiparous (n = 48) and multiparous animals (n = 111). These cows were inseminated based on estrous expression, which was monitored by qualified personnel using indicators such as standing behavior, vocalization, increased number of steps, social interaction, and other behavioral signs.

**Fig. 1:**
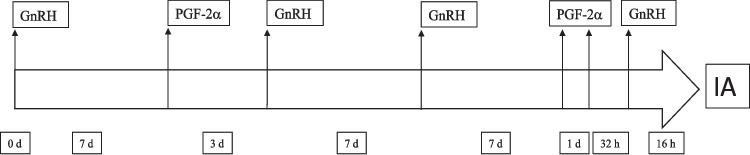
Diagram of the modified double ovsynch synchronization protocol used in lactating Holstein cows at 64 ± 5 DIM

The modified double ovsynch protocol (Herlihy et al. 2012) typically involves two rounds of ovsynch treatments. The first ovsynch protocol begins with the administration of a GnRH analogue (2 ml dose) on day 0. Seven days later, an injection of prostaglandin F2α (PGF2α) (2 ml dose) is administered, and two days after the PGF2α injection, another dose of GnRH is given to induce ovulation of the largest follicle. Seven days later, a second modified ovsynch protocol is initiated; cows receive GnRH treatment followed by PGF2α treatments administered 7 days later, with the final GnRH treatment administered 56 h later, followed by TAI 16 h later (Fig. 1).

The GnRH (0.1 mg/dose gonadorelin diacetate tetrahydrate, Cystoreline) is from Ceva Animal Health, S.A., Barcelona, Spain, and PGF2α (0.15 mg/dose D-Cloprostenol sodium, Dalmazin) is from Fatro, S.p.A., Bologna, Italy. Four high-fertility sires were selected for artificial insemination, and their semen was randomly assigned for representation across seasons. Cows were inseminated by the same veterinarian.

### Data collection

The first group, G-A (n = 63) was inseminated once the modified double-ovsynch protocol was established, while 30 cows were inseminated in winter and 33 cows in summer season. The second group, G-B (n = 96) consisted of inseminated cows not subjected to any synchronization treatment. Cows were visually monitored daily in the morning and evening after reaching 64 ± 5 DIM for signs of behavioral estrus by trained personnel. Gaude et al (2021) identified the most reliable visual estrous indicators besides standing behavior, including increased activity and participation in estrus groups, mounting group members, and the resting of the head on the backs of other cows. Cows detected in estrus based on these signs in the morning were inseminated in the evening, and vice versa, following the standards of breeding practices.

The following data were collected: a) measurement of individual rectal temperature for all cows using a UT-103 digital thermometer (A&D Instruments Limited); b) evaluation of the quantity of viscous mucus discharge from the vulva, using the following scale: 1 (copious), 2 (moderate), 3 (absent) based on Deo and Roy’s classification (Bernardi et al. 2016). The scale for quantifying vaginal discharge was established through a subjective visual assessment, performed by a single observer to avoid inter-observer variability. Vaginal stimulation was conducted transrectally, observing the amount of vaginal discharge that fell to the ground. The animals with the most and least discharge were used as reference extremes, while the others were categorized into the three categories explained above based on their visual proximity to these extremes.

The uterine tone was subjectively graded by transrectal palpation, with only two possible values:'1'(maximum tone, turgid uterus) or'2'(minimum tone, flaccid uterus) (Bonafos et al. 1995). These values were consistently assigned by the same veterinarian to avoid inter-observer variability.

Measurements for endometrium thickness (1), and uterine thickness, including endometrium, myometrium, and perimetrium (2), as well as the length, and width of the preovulatory follicle, were objectively measured in (cm) using trans-rectal ultrasound (MyLabOneVet, Esaote SPA © with a 7.5 MHz transductor). Blood extraction was performed to measure serum progesterone concentration, as explained in Sect."Determination of progesterone concentration in serum.".

### Determination of progesterone concentration in serum

Before insemination, blood samples were collected only in cows via puncture of the median caudal blood vessels into 4 mL evacuated serum collection tubes (BD™ Vacutainer™ Serum Tubes, Madrid, Spain). After collection, blood samples were refrigerated until centrifuged (20 min at 1,600 × g; 4 °C). Serum was stored at − 20 °C until assayed for progesterone (P4) concentrations using an enzyme immunoassay for the quantitative determination of P4 in serum with a commercial progesterone ELISA kit (Progesterone ELISA EIA-1561, DRG Instruments GmbH, Marburg, Germany) following the manufacturer’s instructions. Each sample was assayed in duplicate, and the mean value of both measurements was used. The detection limit for P4 ranged from 0 to 40 ng/mL, with an analytical sensitivity of 0.045 ng/mL. Optical densities were measured using a micro-plate reader (Multiskan EX, Thermo Fisher Scientific Inc., Waltham, MA, USA).

From a total of 227 Holstein cows, 159 first and second-parity animals were selected for the first experiment. Randomly, 63 cows were subjected to a modified double-ovsynch synchronization protocol, while 96 cows were inseminated based on observed estrous. Of these 159 animals, 82 were studied during winter and 77 during summer.

### Climate data

The ambient temperature and the relative humidity of the study farm were recorded from January 1 st to March 31 st and from July 1 st to September 30th using a Multi-Use Compact PDF Temperature and Humidity USB Data Logger device (Multicomp Pro, Farnell Components SL, Madrid, Spain). The device was installed at a height of two meters located in the separation aisle between both lactation zones of the farm, sufficient to keep out of cows’ reach. These devices can measure ambient temperatures (T^a^) from −30 °C to + 70 °C with an accuracy of ± 0.5 °C and relative humidity (RH) from 100% with an accuracy of ± 3%. Data were recorded every hour and were used for calculating the THI (Temperature-Humidity Index) using the formula (Mader et al. 2006):

$$\text{THI}=0.81\times {\text{T}}^{\text{a}}+\text{RH}\div 100\times \left({\text{T}}^{\text{a}}-14.4\right)+46.4$$

This formula was selected for this study based on its application in various research studies (Mader et al. 2006; García-Ispierto et al. 2007; Ioannis et al. 2021; Kic 2022) and its suitability for the collected data, which includes dry bulb temperature and relative humidity.

Maximum THI was calculated at 0 days (the day of AI) at 8 days post-AI (uterine embryo implantation) and 16 days post-AI (maternal recognition of gestation).

### Pregnancy diagnosis

Pregnancy diagnosis for all cows was conducted 32 ± 3 days after artificial insemination (AI) using a portable ultrasound scanner (Easi-Scan, BCF Technology Ltd., Livingston, UK) equipped with a 7.5-MHz linear-array transducer. A cow was confirmed pregnant if the developing embryo, a heartbeat, and the presence of a corpus luteum in the ovary on the same side as the corresponding uterine horn were visible. For cows diagnosed as pregnant during this initial examination, pregnancy was reconfirmed 63 ± 3 days after AI using the same ultrasound equipment and transducer.

### Statistical analysis

A Chi-square analysis was performed to compare fertility rates (*pregnant vs non-pregnant animals*) between the hormonally synchronized and non-synchronized groups in each season separately (*summer vs winter*). This test allowed us to determine whether there were significant differences in the mean fertility rates between the groups under study.

The different data collected from G-A have been considered in logistic regression analysis to study their influence on P/AI. The different variables studied in previous AI were individual rectal temperature, the quantity of viscous liquid discharge from the vulva (1 copious, 2 moderate, 3 absent), uterine tone 1 (turgid) and 2 (flaccid uterus), endometrium thickness (cm), uterine thickness (cm), serum progesterone concentration (ng/mL), maximum THI values at AI day, 8 post AI days and 16 post AI days.

To obtain the optimal model, we iteratively removed and added each of the different independent variables under study, while monitoring the Akaike´s Information Criterion (AIC) values until the lowest value was achieved. To analyze the factors influencing the probability of successful artificial insemination (P/AI), the model was represented by a logistic regression model. The model is represented by the following equation:

$$\begin{array}{c}PP\left(P/AI=1\vert X\right)=\\\frac1{1+e^{-\left(\beta_0+\beta_1\cdot\mathrm{Temp}+\beta_2\cdot\mathrm{Discharge}+\beta_3\cdot\mathrm{Tone}+\beta_4\cdot\mathrm{EndoThick}+\beta_5\cdot\mathrm{UterineThick}+\beta_6\cdot\mathrm{Prog}+\beta_7\cdot\mathrm{THI}\_\mathrm{AI}+\beta_8\cdot\mathrm{THI}\_8+\beta_9\cdot\mathrm{THI}\_16\right)}}\end{array}$$

In this equation:

- Temp: stands for the individual rectal temperature.
- Discharge: refers to the amount of viscous liquid discharge from the vulva, categorized as 1 (copious), 2 (moderate), or 3 (absent).
- Tone: indicates the uterine tone, classified as 1 (turgid) or 2 (flaccid).
- Endo Thick: is the thickness of the endometrium in centimeters.
- Uterine Thick: represents the uterine thickness in centimeters.
- Prog: denotes the serum progesterone concentration in ng/mL.
- THI_AI, THI_8THI_8, and THI_16THI_16 represent the maximum Temperature-Humidity Index (THI) values on the day of artificial insemination, 8 days after artificial insemination, and 16 days after artificial insemination, respectively.
- THI_AI: represents the maximum Temperature-Humidity Index (THI) values on the day of artificial insemination.
- THI_8 represents the maximum THI values 8 days after artificial insemination.
- THI_16 represents the maximum THI values 16 days after artificial insemination.

The odds ratio values were computed by exponentiating the logistic coefficient corresponding to the reference category. These analyses were conducted using the R statistical package (version 4.2.2; R Core Team 2022).

The Wilcoxon test was used to examine the disparities in maximum THI and serum progesterone concentration between winter and summer periods. Significant results were considered at *P* < 0.05 and a trend when 0.05 < *P* ≤ 0.10.

## Results

### Relationship between THI and cold/warm months

**Fig. 2:**
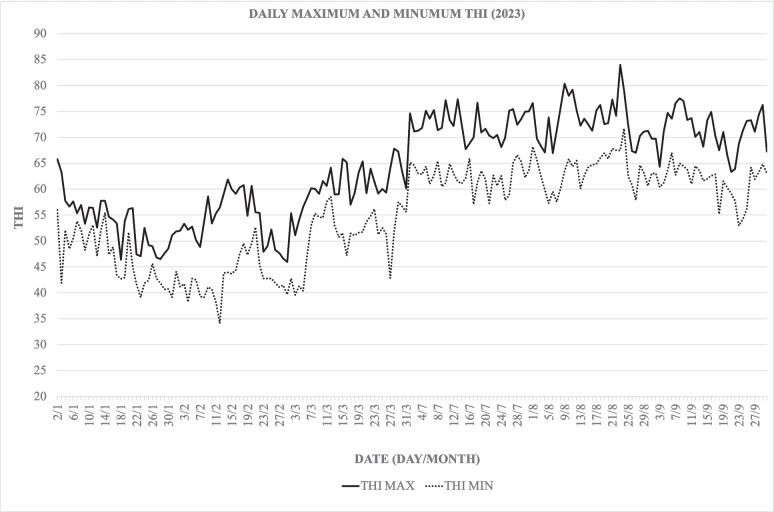
Common to both experiments: THI during the months of the study, the maximum THI is shown as a solid line and the minimum THI is shown as a dashed line

The maximum and minimum THI values recorded during the study months are represented in Fig. 2 showing that during the warmer period, there was a significant increase (*p* < 0.01) in the maximum and minimum THI compared to the colder months.

The minimum THI value recorded at the beginning of the year was 34.10 on February 12th, while the maximum THI value of 67.83 was reached on March 28th. Additionally, during the warm season, the maximum and minimum THI values (83.95 and 63.35) were recorded on August 23rd and September 21 st, respectively.

**Fig. 3:**
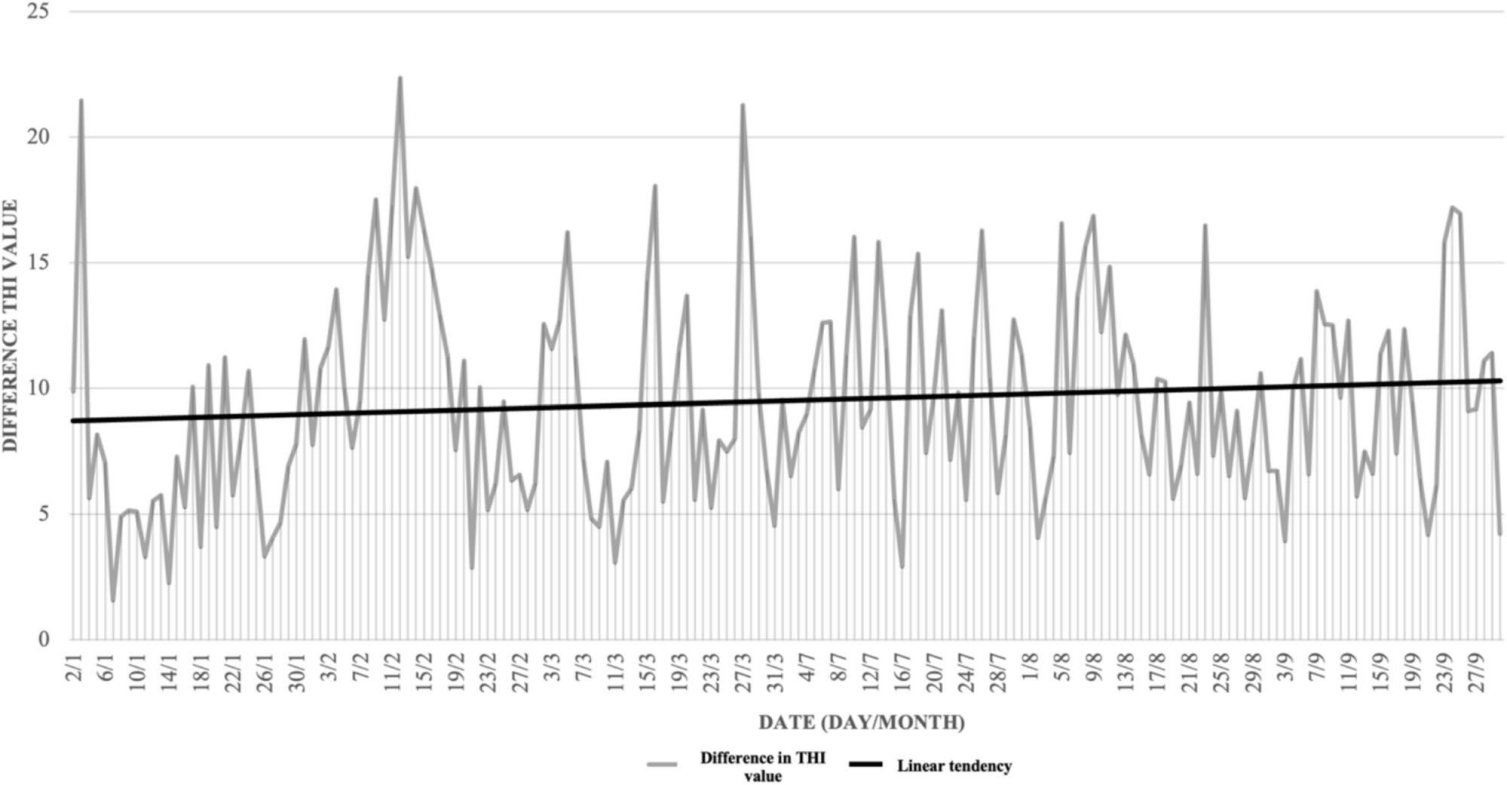
Common to both experiments: Difference value between maximum and minimum daily THI over the study period

Moreover, the difference between the maximum and minimum THI values was calculated and is represented in Fig. 3.

The Temperature-Humidity Index (THI) classification according to Armstrong (1994) is as follows:

- **THI < 72:** No heat stress or minimal stress.
- **THI 72–78:** Mild heat stress.
- **THI 79–88:** Moderate heat stress.
- **THI > 89:** Severe heat stress.

Daily temperatures and humidity levels in the cold months remained constant throughout the day compared to the warmer season. In this last period, there was a greater daily variation in temperature and humidity. Heat stress (THI ≥ 72), specifically mild heat stress (Armstrong 1994) was recorded for 13 days in July, 18 days in August, and 14 days in September, with a higher average duration of 7.2 h in August compared to July and September, which did not exceed 4 h, as represented in Table 1.

**Table 1:** The number of days and mean of hours with mild stress (THI 72 ≥ x ≤ 78) and moderate stress (THI 79 ≥ x ≤ 88) were recorded during the warm months (July, August and September) in which the study was conducted

Month	Nº days with THI 72 ≥ x ≤ 78	Nº days with THI 79 ≥ x ≤ 88	Mean of hours a day with THI 72 ≥ x ≤ 78	Mean of hours a day with THI 79 ≥ x ≤ 88
July	16	0	5,688	0
August	19	4	8,158	3,75
September	14	0	4,857	0

Table 2 reports statistically significant differences between the hormonally synchronized and non-synchronized groups. In the pooled data analysis, a t-statistic of 1.896 (*P*-value = 0.05), irrespective of the season. The synchronization protocol demonstrated a trend of better results in the summer (P-value = 0.091).

**Table 2:** Chi-square and *P*-value comparisons between hormonally synchronized and non-synchronized groups in different seasons

Comparison	n	Chi-square	*P*-Value
All data (Synchronized vs non-synchronized)	159	1.896	0.050*
Winter (Synchronized vs non-synchronized)	82	0.957	0.341
Summer (Synchronized vs non-synchronized)	77	1.707	0.091

*Summer* July, August, and September; *Winter* January, February, and March; *Synchronized* animals submitted to a modified double-ovsynch protocol; *non-synchronized* animals not submitted to any ovulation synchronization protocol; *N* number of animals; ^*^P-value = 0.05 statistically significant difference

In Table 3, the hormonal synchronized group in the summer exhibited a higher proportion of pregnant animals (48,5%) compared to the non-synchronized group (29,5). Similar results were obtained during the winter season, with synchronized animals actively having higher fertility (43,3%) compared to those inseminated based on observed estrous (32,7%). It should be noted that the highest percentage of not pregnant cows was observed during summer in cows inseminated based on observed estrous (70,5%).

**Table 3:** Fertility distribution by season and synchronization protocol in dairy cattle

Season	Synchronization	Fertility	Animals (n)	%	*P*-value
Summer	Synchronized	-	17	51.5	> 0.1
		+	16	48.5	
	Non-Synchronized	-	31	70.5	> 0.1
		+	13	29.5	
Winter	Synchronized	-	17	56.7	> 0.1
		+	13	43.3	
	Non-Synchronized	-	35	67.3	> 0.1
		+	17	32.7	
Total animals	Synchronized	-	34	53.9	> 0.1
		+	29	46.1	
	Non-Synchronized	-	66	68.8	> 0.1
		+	30	31.2	

*Summer* July, August, and September; *Winter* January, February, and March; *Synchronized* animals submitted to a modified double-ovsynch protocol; *Non-Synchronized* animals not submitted to any ovulation synchronization protocol; **(**-) means not pregnant at first insemination; (+) means pregnant at first insemination; *%:* percentage of pregnant or not pregnant animals of each group. The chi-square test did not reveal significant differences between the groups in terms of fertility during winter. This suggests that, in this instance, hormonal synchronization did not have a significant impact on pregnancy rates compared to non-synchronized animals during this season

**Fig. 4:**
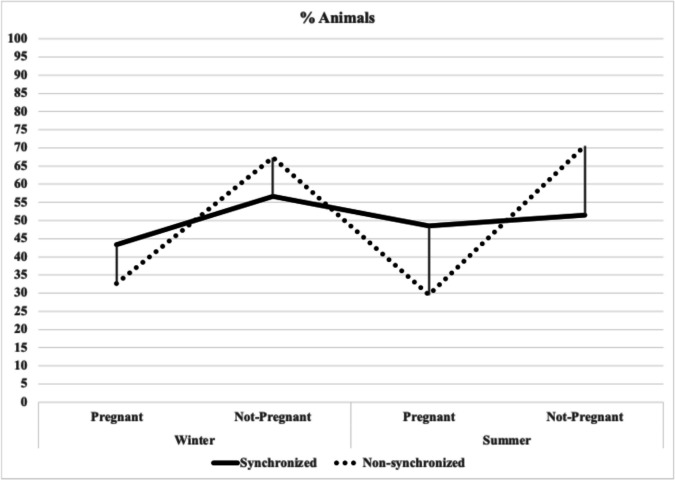
Experiment 1: Lineal graph representing the percentage of inseminated animals: fertility comparison by synchronization and season

Table 3 data are represented graphically in Fig. 4, showing that pregnancy rates remain stable across seasons in animals synchronized with the modified double-ovsynch protocol, underscoring the efficacy of this synchronization method. Furthermore, fertility rates in synchronized animals were higher than in non-synchronized counterparts during summer, suggesting that this protocol can sustain fertility even under heat stress. This difference was only numerical (*P* > 0,05). Conversely, non-synchronized animals exhibited marked seasonal variability in pregnancy rates, with consistently lower fertility irrespective of season.

### P4 concentration at the AI time, during the study

**Fig. 5:**
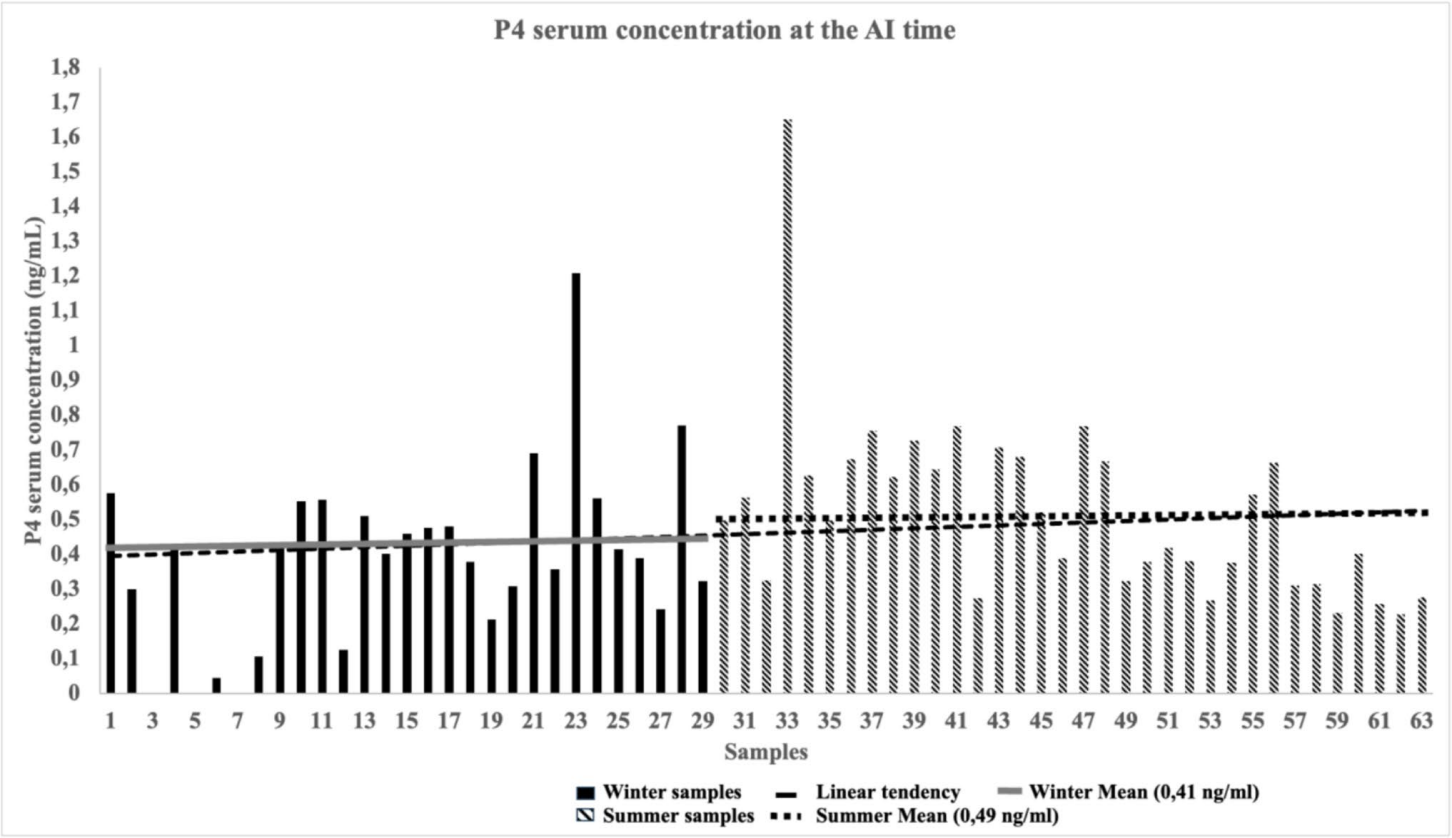
Serum progesterone concentrations (ng/mL) for samples collected from cows synchronized with double-ovsynch at the time of AI in cold (0–30) and warm (31–63) periods

Cows were sampled at the time of insemination to determine serum progesterone concentration. Results represented in Fig. 5, showed an increase in progesterone concentration (*P* = 0.035) in cows sampled during the warm period compared to cows initially sampled during the cold season.

The mean of the first 30 samples from cows synchronized in cold months was lower than the other 34 samples collected during the warm season (0.41 ng/mL ± 0.023 ng/mL and 0.49 ng/mL ± 0.023 ng/mL) respectively. This difference was statistically significant (*P* < 0.05).

### Effect of different variables studied on P/AI

From the 63 cows evaluated at the time of AI, several quantitative variables were recorded, as shown in Table 4, and their relationship with pregnancy diagnosis at first insemination was studied. There were no significant differences between pregnant and non-pregnant lactating Holstein cows.

**Table 4:** Descriptive statistics (mean ± standard deviation) for quantitative variables such as rectal temperature, endometrium and uterine thickness, length and width of the dominant follicle, progesterone, THI maximum on the day of AI, at 8- and 16-days post AI of 63 cows according to pregnancy diagnosis at first insemination ((-) no pregnancy and, (+) pregnancy)

Variable	Pregnancy/AI	n	Mean ± standard deviation
Rectal temperature	-	34	38.32 ± 0.37
	+	29	38.2 ± 0.35
Endometrium thickness	-	34	1.00 ± 0.97
	+	29	1.10 ± 1.07
Uterine thickness	-	34	1.26 ± 1.24
	+	29	1.31 ± 1.26
Length and width/follicle	-	34	1.09 ± 1.03
	+	28	1.13 ± 0.99
Progesterone	-	34	0.283 ± 0.032
	+	29	0.279 ± 0.035
THI maximum at 0 d/AI	-	34	63.06 ± 10.19
	+	29	65.07 ± 71.21
THI maximum 8 d/AI	-	34	62.8 ± 10.49
	+	29	65.54 ± 9.2
THI máximum 16 d/AI	-	34	64.93 ± 0.2
	+	29	67.7 ± 0.22

Two subjective qualitative variables were considered in this study such as uterine tone and the quantity of viscous mucus discharge from the vulva. Turgid uterine and copious viscous liquid discharge from the vulva was presented in most cows incorporated in this experiment (n = 58). Only 6 cows showed flaccid uterine and little viscous mucus discharge from the vulva at the time of data collection during the study.

### Relation of the different variables and THI at days 0, 8 and 16 post AI with P/AI in cows subjected to a synchronization protocol.

This study examined the impact of the THI at three key points: at the time of artificial insemination (AI), 8 days after AI, and 16 days after AI. The analysis included both qualitative and quantitative variables recorded at the time of AI and explored how these factors might interact with pregnancy outcomes following the first insemination (Table 4). To achieve this goal, a logistic regression analysis was done among all the variables studied the statistical by three variables that showed an association with P/AI: rectal temperature, minimal uterine tone, and length and width of the dominant follicle (Table 5).

**Table 5:** Logistic regression model of the quantitative and qualitative variables studied in lactating Holstein cows to explain the variable pregnancy diagnosis at first insemination. Estimate std (standard deviation), P (probability), OR (odds Ratio), CI 95% (95% confidence interval)

Variable	Estimate Std	Z-value	P value	OR	IC 95%
Rectal temperature	−1.498	−1.771	0.076	0.224	0.038–1.089
Uterine tone (flaccid uterus)	−1.787	−1.555	0.119	0.107	0.008–1.181
Length and width/follicle	−1.879	−2.762	0.005^**^	0.162	0.041–0.555

^**Significance level*P* <0.01^

As a result of the statistical analysis, it is evident that the maximum THI at 0 d before insemination and at 8 days and 16 days post-AI did not affect the variables and P/AI. However, an increase in follicle length and width implies a higher probability of about 0.162 times no gestation at first AI. The other two variables, although they don´t have a significant association with P/AI, are necessary to build the statistical model. Rectal temperature and minimum uterine tone negatively correlate with P/AI (*P* = 0.07 and 0.11 respectively).

## Discussion

The results obtained in this study showed a THI higher than 72 in an oceanic climate (classified as mild stress) (Armstrong 1994) with consequences on reproductive function. This elevated index was not sustained throughout the entire day. Brügemann et al. (2012) noted that climate change could subject livestock to temperatures and humidity levels outside their comfort zone, even in areas not traditionally associated with extreme climatic conditions. This study was motivated by the need to determine whether these regions are beginning to present stressful conditions that impact livestock productivity. The primary objective was to assess if these climatic changes are becoming a concern for animal welfare and production, and to explore mitigation strategies before they become a significant issue.

When selecting a method to interpret the THI, various studies prefer to use the daily mean of THI (Schüller et al. 2017; North et al. 2023) arguing that this approach offers a complete evaluation, including the periods where the animal is experiencing heat stress and the periods with thermal comfort. However, De Rensis et al. (2015) suggest that using the maximum daily THI may offer a more realistic option for thermal stress conditions in cattle. This suggestion is supported by several studies (Ravagnolo and Misztal 2000; García-Ispierto et al. 2006, 2007; Bernabucci et al. 2014; Santolaria et al. 2010), which demonstrate that it effectively identifies thermal stress conditions.

Our study showed an increase in THI during the warmer months of July, August, and September, reaching values deemed by various to be sufficient to induce heat stress in dairy herds (≥ 72) (Armstrong 1994; da Costa et al. 2015; Moretti et al. 2017). Throughout most of the summer, heat stress in this area was classified as mild, with THI values primarily between 72 and 78 (Armstrong 1994). In August, there were four days when heat stress levels reached values within the range for moderate heat stress (THI between 79 and 88) on the heat stress scale. In high-production dairy cows, various studies confirmed heat stress begins at 72 to a decreased conception rate (McGowan et al. 1996; Morton et al. 2007; Schüller et al. 2017).

This finding is noteworthy because oceanic climates are characterized by having a mean temperature of 0 °C or higher during the coldest month, unlike continental climates, where the mean temperature in the coldest month typically falls below 0 °C. Summers in oceanic climates are generally mild, with the warmest month averaging below 22° C, meaning they are warm but not excessively hot (Scholarly Community Encyclopedia 2022).

The use of exogenous hormones to synchronize a new follicular wave and enable timed artificial insemination (TAI) is a recognized strategy to mitigate heat stress-induced infertility in dairy cows (Roth et al. 2001a, b). The double-ovsynch protocol, which uses hormonal treatments to time ovulation precisely, has shown the potential to maintain consistent fertility across seasons, especially under heat stress. Our study, the statement is represented where synchronized cows in summer had higher pregnancy rates (48.5%) compared to non-synchronized cows (29.5%) numerically, but not statistically significant (*P* > 0.05).

Comparisons of double-ovsynch and presynch-ovsynch protocols have previously shown that double-ovsynch increases pregnancy rates per first insemination, especially in first-parity cows (65.2% *vs* 45.2%, *P* < 0.05), while results for multiparous cows were similar (37.5% *vs* 39.3%) (Souza et al. 2008). These results align with our findings, where synchronized cows generally achieved higher fertility rates than those inseminated based on observed estrous, in both summer and winter.

A notable advantage of the double-ovsynch protocol is its ability to increase progesterone (P4) levels before administering PGF2α, which can enhance conception rates at the first insemination. Higher P4 levels during initial GnRH injection correlate with improved fertility, and better oocyte quality (Chebel et al. 2006; Stevenson and Pulley 2012). By boosting P4 through presynchronization, the double ovsynch protocol may improve conception rates when temperatures rise, as noted by Roth et al. (2001a, b) and Ayres et al. (2013). These benefits are particularly relevant under heat stress, which lowers P4 levels and reduces estrous expression, highlighting the protocol’s value in summer. Cooling methods, such as fan systems, also help improve estrous behaviour and P4 serum levels in lactating cows during hot weather.

In winter, our study found no significant differences in fertility between synchronized and non-synchronized cows, suggesting that double-ovsynch is especially effective in heat-stressed environments (Roth et al. 2001a, b; Ayres et al. 2013). Figure 4 illustrates the protocol’s ability to stabilize pregnancy rates across both seasons, underscoring its potential to reduce seasonal fertility fluctuations. These results support the modified double-ovsynch protocol as a viable tool for maintaining stable fertility, even under heat stress. These findings align with studies by Roth et al. (2001a, b) and Ayres et al. (2013), which show that hormonal synchronization and increased P4 levels can enhance reproductive success. Double-ovsynch thus emerges as a valuable strategy to minimize the effects of seasonal temperature alterations on dairy cow fertility.

Cows sampled during warmer periods exhibited higher progesterone concentrations than those sampled during the cold periods. Several studies have reported varying P4 serum concentrations in summer compared to winter (Wilson et al. 1998; Roth et al. 2000), which means that any alteration of serum P4 can be explained by different authors.

The effects of heat stress on plasma P4 concentrations have been hypothesized to be associated with several causes such as luteal blood flow alteration, and dry matter intake (Rensis and Scaramuzzi 2003). However, a higher serum P4 concentration has been observed in cows exposed to acute heat stress, while chronic heat stress tends to result in lower P4 levels In this study, the animals were likely experiencing acute heat stress, though the double ovsynch protocol may also contribute to increased P4 levels (Roth et al. 2001a, b; Ayres et al. 2013). Despite the increased serum P4 concentration during warmer sampling periods, no relationship was observed between serum P4 and P/AI.

Shehab-El-Deen et al. (2010) and Ferreira et al. (2016) have pointed out that the main cause of fertility decline in summer is due to a reduction in the quality of follicles. The decrease in follicle quality is due to altered hormone production leading to alterations at the plasma and nuclear level and decreased follicle size (Schüller et al. 2017). The statistical analysis showed that an increase in follicle size is associated with a decrease in P/AI. This is because the animals are synchronized, ensuring that the follicle size is optimal for ovulation. However, if the follicle grows too large, it becomes overmature, leading to a lower P/AI. However, a direct relationship with THI has not been demonstrated.

Variables such as endometrial thickness, uterine thickness and uterine tone were also examined. Only the absence of uterine tone was significantly associated with a decrease in the number of pregnancies per the first AI.

The maximum THI on the day of the insemination, 8 days post-AI (of embryo implantation), and 16 days post-AI (maternal–fetal recognition) were measured due to their relevant impact on the pregnancy period. However, our study didn’t show any interaction between these variables and P/AI.

In some oceanic climate regions, few mitigation strategies are implemented to reduce heat stress effects on dairy cattle, likely due to its limited perceived relevance until now. Some farms have fans in resting areas, but the majority lack mitigation strategies. Dash et al. (2016) proposed 4 points to mitigate heat stress: the development of genetically heat-tolerant dairy cows, increased nutrient density, environmental modification, and the use of TAI protocols. The most common protocol used is the ovsynch. This protocol can successfully synchronize ovulation and increase the conception rate (Hoque et al. 2014). Protocols such as CIDR synch and presynch have also improved the conception rates and P/AI in Holstein cattle during heat stress (El-Tarabany and El-Tarabany 2015). Dirandeh et al. (2015) indicated that the double-ovsynch protocol is more efficient than the rest of the hormonal synchronization protocols for TAI. In this study, using the modified double-ovsynch synchronization protocol mitigated most heat stress effects.

## Conclusions

This study aimed to determine whether the temperature and humidity index (THI) reached stress-inducing levels for dairy cattle under oceanic climate conditions and to analyze fertility rates and reproductive parameters in cattle subjected to hormonal synchronization compared to those not synchronized, across both winter and summer seasons. The results obtained provided substantial evidence to support these objectives, particularly in quantifying heat stress and demonstrating the efficacy of the synchronization protocol, although a direct association between THI and P/AI was not established..

Based on THI measurements and quantification, mild heat stress was observed in the lactating herd during the summer months of the study (July, August, and September) in the present oceanic climate region. The modified double-ovsynch protocol improved pregnancy rates in Holstein cows under summer heat stress. Pregnancy rates remained consistently higher in synchronized cows across seasons, highlighting the protocol’s effectiveness in stabilizing fertility despite seasonal temperature variations. Elevated THI levels negatively impacted physiological variables related to reproductive uterine and ovarian function and altered progesterone hormone levels. However, no direct association with P/AI was identified in this study.

## Data avalilability

The data utilized in this article will be made available upon reasonable request from interested parties.

## Declarations

### Recommendations for a further studies

Future studies should consider evaluating the modified double-ovsynch protocol in different climatic regions and under varying management conditions to assess its generalizability. Additionally, further research should focus on the underlying physiological mechanisms linking THI, hormonal fluctuations, and reproductive outcomes, providing a deeper understanding of the interaction between heat stress and reproductive efficiency. Expanding the sample size and including different breeds could also enhance the applicability of the findings.
